# Development of LC-MS/MS and GC-MS/MS Methods for the Detection of Ethyl Glucuronide (EtG) and Ethyl Palmitate (EtPa) in Hair

**DOI:** 10.3390/molecules30132681

**Published:** 2025-06-21

**Authors:** Sharnette Ashiru, Ethan Webster, Benjamin Barrett, Mathew Wade, Brian Rooney

**Affiliations:** 1 AttoLife Limited, 33 Scottow Enterprise Park, Lamas Rd., Badersfield, Norwich NR10 5FB, UK; s.ashiru@attolife.co.uk (S.A.); e.webster@attolife.co.uk (E.W.); m.wade@attolife.co.uk (M.W.); 2 School of Life Sciences, Pharmacy and Chemistry, Kingston University, 55-59 Penrhyn Rd., Kingston upon Thames KT1 2EE, UK; b.barrett@kingston.ac.uke

**Keywords:** ethyl glucuronide (EtG), ethyl palmitate (EtPa), hair testing, method validation

## Abstract

Alcohol abuse is a widespread addiction globally, leading to long-term health issues and social consequences. Ethyl glucuronide (EtG) and ethyl palmitate (EtPa) are frequently requested by local authorities, solicitors, or private individuals to assess long-term chronic excessive alcohol consumption. In this paper, we present a validation process aimed at developing sensitive methods for detecting EtG and EtPa in hair samples. EtG was extracted by overnight sonication in water followed by sample clean-up using solid phase extraction (SPE) and analysis by liquid chromatography–tandem mass spectrometry (LC-MS/MS). EtPa was extracted using a simple ultrasonication extraction followed by analysis using gas chromatography–tandem mass spectrometry (GC-MS/MS). The analytical method was validated by assessing linearity, precision, accuracy, recovery, sensitivity, and selectivity. Both EtG and EtPa methods obtained a coefficient of determination (r2) above 0.999 across concentration ranges of 4, 8, 16, 24, 48, and 96 pg/mg and 120, 240, 360, 480, 600, and 720 pg/mg. Extraction recoveries were both close to 100% with stable retention times and proven sensitivity and selectivity. These methods were validated according to the standards set by the United Kingdom Accreditation Service (UKAS) Lab51 and ISO 17025.

## 1. Introduction

Alcohol abuse is one of the most common addictions in the world, causing long-term health and psychosocial implications [1]. Traditionally, alcohol abuse has been evaluated by measuring biomarkers of liver function [2]; however, this approach is not correlated with alcohol intake, as other drugs and toxic substances may result in hepatic injury [3]. A more specific approach to measuring alcohol consumption is to measure the concentration of the biomarkers that are formed in the direct presence of ethanol [4]. Some of these markers in the hair include ethyl glucuronide (EtG) and ethyl palmitate (EtPa) [5]. The testing of EtG and EtPa is frequently requested within legal proceedings to provide a long-term assessment of a person’s alcohol consumption. These tests are required by local authorities, solicitors, or private individuals who may be seeking alcohol tests independently of legal proceedings. Their application ranges from ensuring compliance with court orders to protecting child welfare.

EtG is a stable phase two metabolite produced through glucuronidation following ethanol exposure [6]. Ethyl palmitate is a fatty acid ethyl ester (FAEE) formed through the esterification of fatty acids and ethanol [7]. EtG and EtPa are recognised as diagnostic tools to identify chronic excessive alcohol consumption by the Society of Hair Testing (SoHT) [8]. The 2019 SoHT consensus establishes interpretative guidelines that state that an EtG concentration equal to or greater than 30 pg/mg in a hair segment with a length of 3–6 cm suggests chronic excessive alcohol consumption. Similarly, an EtPa concentration greater than or equal to 350 pg/mg in a 3 cm hair segment or a concentration greater than or equal to 450 pg/mg in a 6 cm hair segment is indicative of chronic excessive alcohol use. This consensus also provides interpretative guidelines for abstinence, where an EtG concentration lower than or equal to 5 pg/mg does not contradict self-reported abstinence [8]. Conversely, for EtPa, a concentration lower than or equal to 120 pg/mg for a 0–3 cm segment or lower than or equal to 150 pg/mg for a 0–6 cm segment does not contradict self-reported abstinence [8]. While the use of LC-MS/MS to measure EtG concentrations in hair is well-established and numerous analytical methods have been developed for this purpose [9], to the best of our knowledge, no new methods have been published in recent years.

Vignali et al., 2018 [10], detailed the difference between using GC-MS/MS and LC-MS/MS to quantify EtG. The most significant difference between the two processes is related to the extraction technique. The sample preparation was time-consuming and although they utilised SPE, this was followed by a 1 h derivatisation step with a reagent that needed to be evaporated before injection on the GC-MS/MS. For this present study, this would not have been a viable option due to the need for high-throughput analysis. Most protocols include SPE sample clean-up for the extraction of EtG; however, the differences between laboratories vary in terms of solvents chosen for extraction, length of ultrasonication, and the choice of SPE cartridges. The sample preparation technique used in this study was optimised to provide the best recovery for EtG, which required a longer ultrasonication time.

A combined method to extract EtG and EtPa was considered by the authors; however, being that EtG and EtPa are very different molecules regarding their behaviour in extraction and chromatography, this was not possible when considering the required sensitivity and the miscibility of the solvents used for each extraction. A combined extraction of EtG and FAEE was developed by Vaiano, F. et al. [11] for the analysis of meconium samples, where the analysis of FAEE was achieved using a C8 column and the analysis of EtG was achieved using a C18 column. This method also utilised solid phase extraction (SPE) and had separate elution steps for EtG and FAEE; however, due to the instrument capabilities of the laboratory and the cost of multiple columns, it was decided to utilise GC-MS/MS. The literature including Pichini, S. et al. [12] presented a method that can be used to quantify EtPa using reversed-phase chromatography on a C8 column, yet due to the hydrophilic nature of EtG, this would prove ineffective at retaining EtG in the same method. The extraction technique implemented for EtPa in this current study was very quick compared to that of EtG, which required the use of SPE in order to achieve maximum sensitivity and selectivity due to the low concentrations required.

Both of these methods met the validation requirements of the UKAS accreditation of laboratories performing analysis on toxicology samples (Lab 51). The United Kingdom Accreditation Service (UKAS) is a national body, recognised by the government, that assesses laboratories to ensure conformity to nationally and internationally agreed standards, including ISO 17025 [13]. Lab 51 is a publication authored by the UKAS, which sets out the requirements of ISO 17025 [14]. This is for laboratories undertaking the testing of drugs and their associated metabolites within forensic services.

The aim of this study was to develop sensitive methods for the determination of EtG and EtPa in hair samples. After full validation, these methods were deployed in the determination of alcohol consumption in case work samples.

## 2. Results

### 2.1. Optimised Transitions for EtG and EtPa

Table 1 provides a summary of the analytes measured using two different mass spectrometry techniques, namely LC-MS/MS and GC-MS/MS. Table 1 details the polarity, precursor and product ion masses, collision energy, retention time, and dwell time for each analyte and its corresponding internal standard.

### 2.2. Linearity and Retention Time Stability

For all validation experiments, the measured R^2^ value was found to be greater than 0.99 for both EtG and EtPa using a linear calibration and 1/x weighting as shown in Table 2.

### 2.3. Precision

Precision was assessed using within-batch precision, between-batch precision, an analysis of incurred samples, and bias. All data obtained for within-batch and between-batch precision produced a coefficient of variation (CV) within the acceptance criteria of 20% as shown in Table 3 and Table 4 below.

To measure the %CV of incurred hair, three separate aliquots of incurred hair were extracted in a single batch, repeated with fresh aliquots over 5 batches to obtain a total of 15 separate replicates. The CV% values of EtG and EtPa obtained were 11.2% and 18.1%, which passes the internally set criteria of a value less than 20%.

### 2.4. Bias

To determine the bias, the percentage deviation was calculated using data from the within-batch precision experiment. For EtPa, the mean biases for the 180, 300, and 500 pg/mg samples were −0.6, −2.3, and −4.9%. Additionally, bias was observed at the two cut-offs for excessive alcohol consumption, 350 pg/mg (3 cm hair sample) and 450 pg/mg (6 cm hair sample), which were 2.99 and 2.69%. The average bias for this method was −0.42%. Bias for EtG was calculated using the two cut-offs, 5 pg/mg and 30 pg/mg, resulting in biases of −8.77 and −2.45%.

### 2.5. Sensitivity and Selectivity

Sensitivity and selectivity were assessed by analysing three separate samples; this included samples of a blank matrix that contained the internal standard only, blank hair samples that were spiked using known concentrations of various drugs, and a sample spiked with an FAEE mixture containing ethyl palmitate, myristate, oleate, and stearate. This was to demonstrate the absence of interfering peaks that would affect the quantitation of EtG and EtPa.

**Figure 1:**
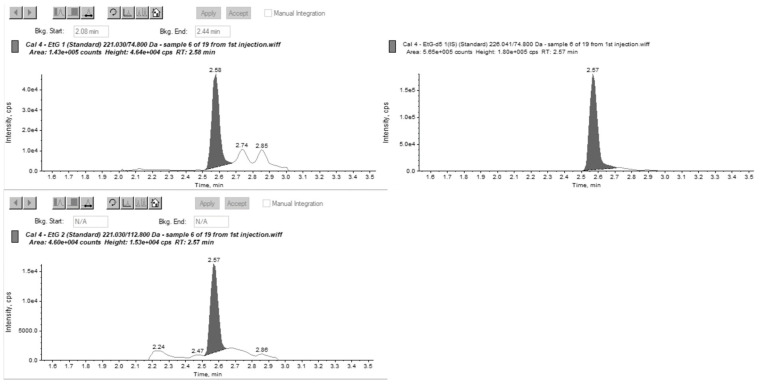
MRM chromatograms for EtG at a mid-level calibration concentration m/z 221.03 → 74.8 quantitation transition (**left**), m/z 221.03 → 112.8 confirmation transition (**bottom left**), and m/z 226.04 → 74.8 internal standard transition (**right**).

**Figure 2:**
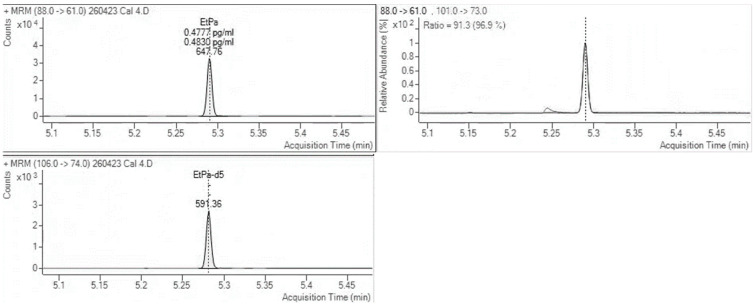
MRM chromatograms for EtPa at a mid-level calibration concentration m/z 88.0 → 61.0 quantitation transition (**left**), m/z 101.0 → 73.0 confirmation transition (**right**), and internal standard transition (**bottom left**) m/z 106.0 → 74.0.

For EtG, two of the blank hair samples had EtG at very low levels, calculated below the cut-off and interpretatively equivalent to abstinence. EtPa was detected at a concentration of 718 pg/mg in the sample containing the FAEE mixture; however, no other interferences were found for both EtG and EtPa. Figure 1 and Figure 2 show MRM chromatographs for mid-level calibration points with good peak symmetry and separation.

### 2.6. Limits of Detection and Limits of Quantification

EtG was detected at a concentration of 2.5 pg/mg, with a signal-to-noise ratio greater than 3:1 for 15 replicates, which was then set as the analytical limit of detection. EtG was tested at 25% of the SoHT cut-off of 1.25 pg/mg, and a consistent S/N could not be maintained at this concentration. The lower limit of quantification, LLOQ, was then set to 4 pg/mg. The LLOQ for EtPa was also set to 120 pg/mg, as shown in Table 5 below.

### 2.7. Recovery

QC samples at concentration levels of 5 and 30 pg/mg (EtG) and 300 and 500 pg/mg (EtPa) were run in extracted and unextracted preparations to compare the peak area ratios, the mean% recovery for these are shown in Table 6 below.

### 2.8. Carryover

As previously described, carryover was assessed by spiking hair samples with EtPa and EtG at concentrations of 720 pg/mg and 500 pg/mg, respectively. Blank solvents were run after these samples, and no carryover was observed for both methods.

### 2.9. Uncertainty

QC and calibrator samples were spiked by different operators, and the extraction of the QC, calibrator, and incurred samples was carried out by different operators over a period of time to incorporate as many variables as possible, i.e., certified reference solutions, extraction techniques, and pipettes. The data used was obtained from the precision experiments and the combined uncertainty was calculated using the QCs at 5 pg/mg for EtG and 180 pg/mg for EtPa alongside data from the incurred samples with a coverage factor of 2, equivalent to 95% confidence. The incorporation of the incurred sample was to show precision in the matrix material. EtG obtained a combined uncertainty of 30.2% (k = 2) and EtPa obtained a combined uncertainty of 56% (k = 2).

## 3. Discussion

This study details a LC-MS/MS method that quantifies EtG and a GC-MS/MS method that quantifies EtPa. These methods meet the analytical requirements of the UKAS Lab 51 and the analytical standard of ISO 17025, a requirement for accredited laboratories performing toxicology testing in civil cases.

The extraction procedure for EtPa showed excellent recovery and sensitivity; this extraction technique is a rapid sonication and precipitation method that provides suitable extracts. For the quantification of EtPa in hair, most sample preparation techniques utilise solid phase microextraction (SPME), followed by GC-MS for quantification [15]; however a much more simplified and cost-effective approach was taken in this study. By contrast, EtG used a SPE technique coupled with hydrophilic interaction liquid chromatography (HILIC). This is an industry standard technique used to retain polar compounds like EtG with enhanced sensitivity. This was a combination that was first publicised by Kintz et al. in 2008 and has remained a common preparation/quantification technique for EtG [16]. This procedure does require longer column conditioning times in order to form a consistent and reproducible layer of water across the stationary phase, which meant that the method was optimised to have a quick elution time. Although modifications could have been made to retain the compound for longer, this would mean sacrificing sensitivity and peak shape, as well as implications for the real-world application of this method, where high-throughput analysis is required. Both extraction methods achieved recoveries close to 100%, ensuring the reported concentration closely matched the actual concentration present in the hair matrix.

Both methods had correlation coefficient R^2^ values greater than 0.99, stable retention times, and proved to be sensitive and selective to the respective analytes, with no interferences present in blank samples or samples spiked with other drug/alcohol mixes.

Our LOD assessment incorporates instrumental performance, sample matrices, and procedural limitations, and both methods achieved good S/N at 2.5 pg/mg for EtG and 60 pg/mg for EtPa. However, the LOQ for EtG was set to 4 pg/mg, which is below the assessed cut-off for abstinence, and the LOQ for EtPa was set to 120 pg/mg.

These methods utilise three quality control concentrations, which are 5, 30, and 80 pg/mg for EtG and 180, 300, and 500 pg/mg for EtPa. These QCs were used to assess the precision of the methods over five batches, all of which obtained %CV values < 10. No carryover was observed in the succeeding blank samples even after concentrations in excess of the highest calibrators. This parameter complies with the requirements of Lab 51 in relation to quantifying concentrations at or greater than the cut-offs for excessive alcohol consumption.

The methodology described in this study compares favourably with other analytical techniques that have been validated to detect these alcohol biomarkers. Tarcomnicu et al., 2010 [17], determined EtG concentrations in hair utilising HILIC-LC/MS-MS. In their study, 25 mg of hair sample was extracted using overnight water/organic incubation followed by 90 min ultrasonication. A five-point calibration curve was prepared, which obtained a R^2^ of 0.992 (*n* = 3). Accuracy and precision were within the limits of 15%, completed on QCs at two different concentration levels, QC Low (80 pg/mg) and QC High (800 pg/mg). Selectivity was tested using blank samples, and no interfering peaks were observed in the chromatograms. Although Tarcomnicu et al., 2010, used less hair than this present study, they utilised a broader calibration range with the LLOQ set to 20 pg/mg versus the current studies’ 4 pg/mg. This is significant for case work applications, as a 20 pg/mg LLOQ will not provide a sufficient quantification of EtG concentrations that determine abstinence or low/moderate alcohol consumption. Tarcomnicu et al., 2010, also did not provide a measurement of uncertainty, another requirement of case work analysis in the UK. By contrast, the uncertainty of our methods was estimated to be 56% for the EtPa method and 30.2% for the EtG method.

A separate validation study by Pirro et al., 2013 [18], used UHPLC-MS/MS to quantitate EtG using 50 mg of hair. Linearity was assessed using a concentration range of 1, 2.5, 5, 7.5, and 10 pg/mg, which covers a lower range compared to that used in this method validation, resulting in an R^2^ of 0.999. Between-batch and within-batch precision was assessed using 10 blank head hair samples spiked at 1, 2.5, and 5 pg/mg. Due to the low concentration range, carryover analysis was not completed. Pirro et al., 2013, validated a within-batch and between-batch %CV of <5% across all concentrations. This method displayed excellent sensitivity at low EtG concentrations but did not provide quantification of the upper ranges consistent with chronic alcohol abuse (30 pg/mg).

Despite the rapid and cost-effective nature of the EtPa extraction technique, there are some noticeable limitations of the method. As there is no sample clean-up, extracts may contain eluents that have the potential to affect GC-MS/MS performance negatively over time and increase maintenance requirements. In addition, EtPa analytical method performance can be affected by the choice of ion source used. The EI source causes extensive fragmentation [19], resulting in smaller fragment ions, with many peaks corresponding to different fragment ions compared to PCI, which is a softer ionisation technique. As mentioned previously, EtPa is part of a group of FAEEs, and EI ionisation produces non-specific fragmentation of FAEE-related compounds.

The EtG and EtPa methods were developed to provide alcohol testing for court-directed and private purposes, where information is required to determine chronic alcohol consumption covering over 3 to 6 months. This is particularly useful for child custody disputes, where child safety is of concern, and legal cases where these methods have since been routinely deployed.

## 4. Materials and Methods

### 4.1. Chemicals, Reagents, and Specimens

EtG calibration and deuterated EtG-d5 internal standard reference standards were purchased from Cerilliant (Round Rock, TX, USA). EtG quality control and deuterated EtPa-d5 internal standard reference standards were purchased from Chiron (Trondheim, Norway). EtPa quality control reference standard was purchased from Restek, and the calibration was from Cole Parmer (Vernon Hills, IL, USA). These reference standards were diluted in methanol and heptane to make the working solutions for EtG and EtPa. Other reagents used include HPLC and LCMS-grade methanol, water, de-ionised water, heptane, acetonitrile, hexane, and 1:1 cyclohexane/heptane—all obtained from Fisher Scientific, Honeywell, and VWR. Blank hair matrix was hair that had been previously donated to AttoLife (Norfolk, UK); the donors confirmed they had not consumed alcohol, and samples were screened to confirm this.

### 4.2. Sample Preparation and Extraction

To prepare the hair, samples underwent a 3-stage washing process. This included the use of water, hexane, and methanol to remove any external contaminants such as hair gel and hairspray. This wash procedure is the same as that in the SoHT 2022 consensus, which states that organic solvents can be used providing the wash procedure is fully validated. Once dried, the samples are then milled to homogenise the hair, and aliquots of 30 mg were weighed out for EtG and 20 mg for EtPa.

The extraction of EtG utilised solid phase extraction (SPE), with samples passed through Biotage Evolute Express AX SPE cartridges (Ystrad Mynach, Wales). Samples of calibration and quality controls were spiked using EtG working solutions and internal standards. All samples were left to sonicate in an ultrasonic bath for approximately 6 h (or overnight) at room temperature in de-ionised water. The cartridges were conditioned using analytical-grade methanol, water, and acetonitrile; then, the addition of the sample extract solution followed. The cartridges were washed using methanol and water; then, hexane was added and left to dry for 15 min under vacuum. EtG was eluted from the cartridges using 2% formic acid in methanol, and the resulting product was dried down and reconstituted in 50:50 acetonitrile/methanol.

The extraction of EtPa was via ultrasonication. After spiking EtPa calibration, QCs and internal standards from working solutions onto the hair samples, 600 µL of 50:50 cyclohexane/heptane was added, and the samples were sonicated for 30 min. The resulting solution was dried down and reconstituted in heptane for analysis.

### 4.3. LC-MS/MS Analysis for EtG

For EtG, analysis was completed using LC-MS/MS on an AB Sciex Exion LC UHPLC coupled to an AB Sciex Mass Spectrometer QTRAP 6500+ (SCIEX, Toronto, ON, Canada). Chromatographic separation was performed using a Pheneomenex Kinetex 2.6 um HILIC 100 Å, LC 100 × 2.1 mm column, and a Pheneomenex SecurityGuard™ ULTRA Cartridges UHPLC HILIC 2.1 mm ID guard column (Torrance, CA, USA). Oven temperature was maintained at 30 °C. A gradient elution was performed with 5 mM ammonium formate in 95:5 water/methanol (mobile phase A) and 95:5 acetonitrile/methanol (mobile phase B) at a flow rate of 0.35 mL/min.

The MS/MS was operated with electrospray ionisation in the negative mode and an ion source temperature of 500 °C. The ion spray voltage was set to −3500 V and to ensure the best sensitivity, the parameters were optimised under multiple reaction monitoring (MRM) modes; the detailed MRM transitions are given in Table 1. An injection volume of 10 µL was used for EtG.

### 4.4. Instrumentation for EtPa

EtPa analysis using GC-MS/MS was conducted on an Agilent 8890 GC system and an Agilent 7000D MS/MS (Agilent Technologies, Santa Clara, CA, USA). To perform separation, a DB-5MS Ultra Inert 20 m × 0.18 mm × 0.18 um column was used with a flow rate of 1 mL/min and an injection volume of 1 µL. The oven temperature programme included an initial temperature of 60 °C with a 50 °C/min ramp to a final temperature of 300 °C and 1.5 min hold. The MS/MS was operated using an EI source at a temperature of 180 °C, and the transitions used to quantify EtPa in each method are shown in Table 1.

### 4.5. Method Validation

Method validation was conducted according to the UKAS LAB 51 and ISO 17025 requirements, including precision, sensitivity, selectivity, recovery, stability, and carryover.

#### 4.5.1. Linearity

Six calibration points were used for each assay; EtG linearity was assessed using calibrators 4, 8, 16, 24, 48, and 96 pg/mg and for EtPa, the linearity was assessed using calibrators 120, 240, 360, 480, 600, and 720 pg/mg. The methods utilised three quality control concentrations distributed over the calibration range; these are 5, 30, and 80 pg/mg for EtG and 180, 300, and 500 pg/mg for EtPa. For each batch, the calibrators and QCs were prepared using freshly spiked samples. A minimum R^2^ value of 0.99 is required in accordance with the UKAS and Lab 51.

#### 4.5.2. Precision

Precision was assessed using within-batch precision, between-batch precision, bias, and the analyses of incurred samples. To determine within-batch precision for EtG, blank hair was spiked at the cut-off concentrations and extracted 10 times within a single batch. The criteria for %CV was a value less than 20%. To assess within-batch precision for EtPa, the same data was used as that to assess the between-batch precision at the same concentrations, except with 5 replicates instead of 10. Between-batch precision included spiking a blank matrix at the three QC concentrations, which were extracted 5 times within a batch on 5 separate days by different analysts. %Bias was calculated using the following calculation:

%Bias = (mean of determinations − true or accepted value) × 100/True or accepted value

#### 4.5.3. Sensitivity and Selectivity

Procedural sensitivity was assessed by determining the limit of detection (LOD) and upper and lower limits of quantification. The lower limit of detection (LLOD) and lower limit of quantification (LLOQ) are determined by repeatedly analysing samples of low concentration. The LLOD is the lowest concentration at which a S/N of at least 3:1 is achieved, and the LLOQ is the lowest concentration at which a S/N of at least 10:1 is achieved. S/N was calculated automatically on the LC-MS/MS using AB Sciex Analyst 1.7.2 software, whereas on the GC-MS/MS, S/N was calculated using MassHunter Quantitation software.

Selectivity and specificity were evaluated by locating a minimum of 6 different sources of alcohol-free material and analysing them according to the extraction method without adding internal standards. Alcohol-free matrix samples with internal standards were also analysed, in addition to samples spiked with a high concentration of drugs outside of the scope of testing for the method. This was to ensure no results were affected by the addition of drugs within the matrix.

#### 4.5.4. Recovery

Extraction recovery for both analytes was evaluated by comparing the peak area of the extracted QC samples with the peak area of the unextracted QC samples. The internal standard performance was assessed by calculating the %CV of the responses of five separately extracted calibration curves; this however has no acceptance criteria and there is no requirement to reach a certain level of recovery for internal standards. The internal standard recovery was also assessed, where the peak area of extracted and unextracted hair samples with internal standards were compared. The criteria for recovery, adapted from the Scientific Working Group for Forensic Toxicology (SWGTOX), required the results to fall within a range of 70 to 130%. There is a lack of standardised extraction techniques for hair testing established by professional bodies. This means that when testing a sample with a “known concentration”, i.e., a proficiency trial, the true value is theoretical and an average of the values given by the participants; thus, the extraction recovery cannot accurately be assessed. Additionally, there is no proficiency trial scheme available to assess EtPa from any known organiser.

#### 4.5.5. Carryover

To assess carryover within the EtPa batches, blank hair was spiked at a concentration of 720 pg/mg, which is the top calibrator. For EtG, carryover was assessed using two different methods, firstly by spiking a sample of blank hair with a concentration of 500 pg/mg, which is 100× the concentration of the cut-off for abstinence, and secondly, by observing the blanks after the top calibration point of 96 pg/mg.

#### 4.5.6. Uncertainty

Uncertainty was calculated using the lowest QC concentration and incurred data from the precision section of the validation. The calculation was performed using the sum of root squares, with a coverage factor of 2, equivalent to 95% confidence.

## 5. Conclusions

In the present study, LC-MS/MS and GC-MS/MS methods were developed for the determination of EtG and EtPa in hair samples. The aim was to support legal professionals in monitoring alcohol consumption using direct and specific alcohol biomarkers, providing an alternative to traditional liver function biomarkers, which can be influenced by several factors (age, gender, comorbidities). This study also aimed to reflect current practices and leverage the enhanced chromatographic capabilities of modern instrumentation. Both methods were fully validated in accordance with the UKAS and Lab 51 guidelines, ensuring their suitability for implementation in any analytical laboratory with confidence in the generation of high-quality results, without the reporting of “false-negative” or “false-positive” results, which could present legal and/or social consequences. This accreditation confirms the efficiency and validity of the analytical processes, supporting the accuracy and traceability of measurements. Moreover, the accredited methods offer commercial advantages, particularly valuable at a time when trust in hair testing within the UK is declining. These techniques present a cost-effective solution for detecting alcohol markers and have already been applied successfully in real legal cases, demonstrating their reliability in the quantification of these biomarkers.

## Figures and Tables

**Table 1:** Optimised MRM transitions and retention times (RTs) for EtG and EtPa.

LC-MS/MS						
Analyte	Polarity	Q1 Mass (m/z)	Q3 Mass (m/z)	Collision Energy (eV)	RT (mins)	Dwell Time (ms)
EtG 1	Negative	221.03	74.8	−20	2.6	250
			112.8	−18		
EtG-d5	Negative	226.04	74.8	−20	2.6	
**GC-MS/MS**						
EtPa	EI	101	73	5	5.29	26.9
		88	61	5		
EtPa-d5		106	75	15		

**Table 2:** Coefficients of determination for analysis of EtG and EtPa. The R^2^ values obtained in all validation batches exceeded the minimum requirement of 0.99 required by the UKAS and Lab 51.

Linearity		
Batch Number	EtG	EtPa
1	0.9976	0.9995
2	0.9945	0.9994
3	0.9992	0.9981
4	0.9997	0.9989
5	0.9995	0.9992
Average	0.9981	0.9990
SD	0.0022	0.0006
CV%	0.22	0.06

**Table 3:** Summary of the between-batch precision of EtG and EtPa from 5 batches.

Between-Batch Precision						
	EtG			EtPa		
Target (pg/mg)	5	30	80	180	300	500
Mean	4.54	29.53	78.69	179	293	475
SD	11.55	1.05	3.76	0.007	0.016	0.023
CV%	3.55	3.55	4.78	3.95	5.47	4.89

**Table 4:** Summary of the within-batch precision of EtG and EtPa from 5 batches.

Within-Batch CV%						
**EtG**						
Batch	1	2	3	4	5	Average CV%
5	5.43	3.19	9.47	8.14	6.09	6.46
30	7.62	2.13	2.96	3.47	2.99	3.83
**EtPa**						
Batch	1	2	3	4	5	Average CV%
180	3.68	5.52	4.04	2.31	1.7	3.95
300	4.49	4.22	3.27	3.87	4.72	5.47
500	4.27	2.68	3.51	3.57	6.07	4.89

**Table 5:** A table showing the signal-to-noise ratio, peak area, and peak symmetry of EtG and EtPa at their respective LLOQ concentrations.

**Analyte**	EtG	EtPa
**Conc (pg/mg)**	4	120
**S/N**	39.2	181.96
**Area**	26,000	2554
**Peak symmetry**	1.76	1.18

**Table 6:** A summary of the results from the recovery experiment at two QC levels—5, 30 pg/mg for EtG and 180, 300 pg/mg for EtPa.

Recovery		
	Mean% recovery low QC (*n* = 5)	Mean% recovery mid QC (*n* = 5)
**EtG**	100.51	125.29
**EtPa**	95.63	95.2

## Data Availability

The datasets presented in this article are not readily available because of privacy concerns.

## Abbreviations

The following abbreviations are used in this manuscript:

EtG	Ethyl glucuronide
EtPa	Ethyl palmitate
GC-MS/MS	Gas chromatography–mass spectrometry
LC-MS/MS	Liquid chromatography–mass spectrometry
QC	Quality control
UKAS	UKAS accreditation of laboratories
LOD	UKAS accreditation of laboratories
LLOD	Lower limit of detection
LOQ	Limit of quantification
SWGTOX	Scientific Working Group for Forensic Toxicology
